# Global panel data on World governance and state fragility from 2006 to 2022

**DOI:** 10.1016/j.dib.2024.110167

**Published:** 2024-02-07

**Authors:** Alejandro Vega-Muñoz, Paloma González-Gómez-del-Miño, Guido Salazar-Sepúlveda

**Affiliations:** a Facultad de Ciencias Empresariales, Universidad Arturo Prat, 1110939 Iquique, Chile; b Public Policy Observatory, Universidad Autónoma de Chile, 7500912 Santiago, Chile; c Facultad de Ciencias Políticas y Sociología, Universidad Complutense de Madrid, 28223 Madrid, Spain; d Departamento de Ingeniería Industrial, Facultad de Ingeniería, Universidad Católica de la Santísima Concepción, 4090541 Concepción, Chile; e Facultad de Ingeniería y Negocios, Universidad de Las Américas, 4090940 Concepción, Chile

**Keywords:** Governance, Cohesion, Economy, Politics, Society

## Abstract

This global panel dataset contains information collected from two different sources (The Fund for Peace and World Bank), on the governance and stability levels of 178 countries between 2006 and 2022. The dataset includes information on 1) Cohesion (security apparatus (C1), factionalized elites (C2), and group grievance (C3)), 2) Economic (economy (E1), economic inequality (E2), and human flight and brain drain (E3)), 3) Political (state legitimacy (P1), public services (P2), and human rights (P3)) indicators, 4) Social and cross-cutting (demographic pressures (S1), refugees and internally displaced persons (S2), and external intervention (X1)), and 5) Governance (voice and accountability (G1), political stability and absence of violence/terrorism (G2), government effectiveness (G3), regulatory quality (G4), rule of law (G5), and control of corruption (G6)). Data analysis was carried out using SPSS version 29 software to ensure a complete description of the data (labels, type and measure of variables, and uniformity of decimals), as well as the imputation possibility of missing data, which will allow future researchers to study both cross-sectional and longitudinal relationships between the five types of indicators and the eighteen indicators reported.

Specifications Table

**Table:** 

Subject	Social Sciences
Specific subject area	Political Sciences, International Relations, and Dynamics of change in Global Society
Data format	Cured, Sorted, Filtered, Analysed
Type of data	Table sav file (SPSS) and Table csv file, Table, and Figure
Data collection	The spreadsheets collected (all in Excel formats) include global information on indicators of security apparatus (C1), factionalized elites (C2), group grievance (C3), economy (E1), economic inequality (E2), human flight and brain drain (E3), state legitimacy (P1), public services (P2), human rights (P3), demographic pressures (S1), refugees and Internally Displaced Persons (S2), and external intervention (X1), voice and accountability (G1), political stability and absence of violence/terrorism (G2), government effectiveness (G3), regulatory quality (G4), rule of law (G5), and control of corruption (G6). These data were collected from The Fund for Peace and World Bank for 178 countries between 2006 and 2022. The data analysis has reflected the blanks for some variables and years, so SPSS software was used to allow imputation of the missing data in the subsequent use of this global data panel. The file is also available in csv format.
Data source location	Coverage of 178 countries with homologated names from both sources. Official data sources: (1) https://fragilestatesindex.org/indicators[1] (2) https://www.govindicators.org[2]
Data accessibility	Repository name: “Data Global Governance and Fragility”, Zenodo Data identification number: 10.5281/zenodo.10080979 Direct URL to data: https://zenodo.org/records/10080979

## 1 Value of the Data

• This dataset brings together a unique set of variables, useful for studying the relationships between the diverse elements that make up state fragility/stability and state governance issues.
• Panel data can be used in cross-sectional and longitudinal time slices to validate and/or extend theories that relate political, economic, and social issues in global society, with an emphasis on state governance and state fragility.
• Social science researchers, especially political scientists, economists and sociologists, and policy makers can benefit from the joint use of both datasets, significantly correlated, and commonly presented in a segregated form, both country and area studies.
• Business decision makers can find value in this dataset, with information that provides a global overview for their international investment decisions.

## 2 Background

This dataset brings together two related topics. On the one hand, State Fragility, an important issue for international development [3], identifies a country with weak state capacity and/or weak state legitimacy [4], which is unable to provide basic functions to a large part of its population [5], as a result of inadequate functioning in aspects of politics, public administration and security as a consequence of poverty, underdevelopment or civil war [6], and which leaves citizens vulnerable to a whole series of disturbances [4].

On the other hand, State Governance manifests itself in a democratic country, encouraging the participation of citizens and businesses in public management [7]. Since democracies establish state governance mechanisms that delimit the political and legal responsibilities of the highest authorities of the country [8]. Considering the effectiveness of state management as a fundamental element to address the challenges and dangers generated by the industrial society [9]. Thus, good governance ensures internal order, prevents social chaos, and avoids the spread of negative effects to other countries [10].

When both topics are studied together, both sets of variables are not considered in an integrated manner. There may be studies of State Fragility with a dataset of Fragile States Index indicators [11], [12], [13]), and Governance Studies with data from the World Bank Worldwide Governance Indicators [14,15]. Thus, the union of both primary datasets [1,2], allows to advance in studies covering new theoretical aspects, given the high correlations between all the variables in the resulting secondary database (see Table 3).

## 3 Data Description

The dataset incorporates eighteen variables/indicators: security apparatus (C1), factionalized elites (C2), group grievance (C3), economy (E1), economic inequality (E2), human flight and brain drain (E3), state legitimacy (P1), public services (P2), human rights (P3), demographic pressures (S1), refugees and Internally Displaced Persons (S2), and external intervention (X1), voice and accountability (G1), political stability and absence of violence/terrorism (G2), government effectiveness (G3), regulatory quality (G4), rule of law (G5), and control of corruption (G6).

The Fragile States Index indicators reported by [16] and [17] were obtained from The Fund for Peace. In the case of the State Governance indicators, the data were collected from the World Bank Worldwide Governance Indicators (WGI) used by [18] and [19]. Both datasets are significantly correlated (see Table 3) but are scarcely studied together [20] and [21]. The details and sources of the variables are given in Table 1.

**Table 1:** Description of the variables in (DATa_Global_Governance_and_Fragility_0622).

Name	Label	Operational definitions	Source	Best historical value (06-22)
1. C1	Security Apparatus	Threats to the security of a State, such as bombings, attacks and deaths in combat, rebel movements, riots, coups d'état or terrorism.	FSI [1]	0.3 (Lowest)
2. C2	Factionalized Elites	Fragmentation of state institutions based on ethnic, class, clan, racial or religious grounds, and confrontation and deadlock among the ruling elites.	FSI [1]	0.7 (Lowest)
3. C3	Group Grievance	Divisions and schisms between different groups in society based on social or political characteristics, and their role in access to services or resources and inclusion in the political process.	FSI [1]	0.3 (Lowest)
4. E1	Economic Decline	Progressive economic decline patterns of society, as measured by per capita income, Gross National Product, unemployment rates, inflation, productivity, debt, poverty levels or business failures.	FSI [1]	1.0 (Lowest)
5. E2	Uneven Economic Development	Inequality within the economy, regardless of the actual economic performance, such as structural inequality based on group (racial, ethnic, religious, or other identity group) or based on education, economic status, or region (urban-rural divide).	FSI [1]	0.5 (Lowest)
6. E3	Human Flight and Brain Drain	Economic impact of human mobility (for economic or political reasons) and its consequences for a country's development.	FSI [1]	0.4 (Lowest)
7. P1	State Legitimacy	Public confidence in state institutions and processes and their effects, given the representativeness and openness of the government and its relationship with the citizenry.	FSI [1]	0.2 (Lowest)
8. P2	Public Services	Basic state functions serving the population, such as the essential services (health, education, water and sanitation, transportation infrastructure, electricity and energy, and Internet and connectivity), and the state's capability to protect its citizens through effective police.	FSI [1]	0.6 (Lowest)
9. P3	Human Rights and Rule of Law	Relationship between the State and the population for the protection of fundamental human rights, observance and respect of freedoms and the generalized non-abuse of individual, group, and institutional legal, political, and social rights.	FSI [1]	0.3 (Lowest)
10. S1	Demographic Pressures	Pressures on the State derived from the demographic dynamics of the population and its environment, related to the vital resources supply (food, access to drinking water and others), health, and those derived from extreme meteorological phenomena and environmental hazards.	FSI [1]	0.7 (Lowest)
11. S2	Refugees and IDPs	Pressure on the State caused by the forced displacement of large communities because of social, political, environmental or other causes. Considering intra-country displacement and refugee flows and recognizing the additional pressure on public services and the humanitarian and security challenges for the host State due to insufficient absorptive capacity and adequate resources.	FSI [1]	0.4 (Lowest)
12. X1	External Intervention	Influence and impact of external actors on State functioning. Whether in security aspects, with covert or overt intervention in the internal affairs of a State at risk affecting the internal power balance, or with economic engagement by external actors creating economic dependence (large-scale loans, development projects or foreign aid, continuous budgetary support, control of finances or management of the State's economic policy). Also considering humanitarian intervention, such as the deployment of an international peacekeeping mission.	FSI [1]	0.3 (Lowest)
13. G1	Voice and Accountability	Citizen perception in a country regarding participation in government elections, freedom of expression, association, and the media.	WGI [2]	2.8 (Highest)
14. G2	Political Stability and Absence of Violence/Terrorism	Perception of political instability and/or politically motivated violence, including terrorism.	WGI [2]	1.6 (Highest)
15. G3	Government Effectiveness	Quality perception of public services and the civil service, and their independence from political pressures, the quality of policy formulation and implementation, and the credibility of the government's commitment to those policies.	WGI [2]	2.5 (Highest)
16. G4	Regulatory Quality	Perception of the government's ability to formulate and implement sound policies and regulations that enable and promote private sector development.	WGI [2]	2.3 (Highest)
17. G5	Rule of Law	Agents' perceptions on trust and compliance with social rules, and in particular the quality of contractual compliance, property rights, the police, and the courts, as well as the likelihood of crime and violence.	WGI [2]	2.1 (Highest)
18. G6	Control of Corruption	Perception of the public power exercise for private benefit, including forms of small and large-scale corruption, as well as the "capture" of the state by elites and private interests.	WGI [2]	2.5 (Highest)

## 4 Experimental Design, Materials and Methods

Data extraction and curation used the following protocol:

1) The data extracted from FSI were presented in 17 annual Excel workbooks (2006–2022) reporting the 12 indicators under study.
2) Some records of 49 countries in the FSI databases had a blank space, which resulted in duplicate labels for these 49 countries.
3) Additionally, 7 countries had different names in different years of FSI, these were homologated as: Israel, Cape Verde, Cote d'Ivoire, Czech Republic, Kyrgyz Republic, North Macedonia, and Slovak Republic.
4) On the other hand, the data extracted from WGI were presented in a single Excel workbook, with the 6 indicators (variables) of interest distributed on separate spreadsheets in this workbook.
5) In the case of these 15 countries, the names presented in both bases were homologated as: Bahamas, Congo Democratic Republic, Congo Republic, Congo Republic, Egypt, Gambia, Guinea Bissau, Iran, Laos, Micronesia, Russia, Sao Tome and Principe, Syria, Turkey, Venezuela, and Yemen. In the case of both Koreas, North Korea and South Korea were chosen.
6) For homogenization of the base in variables by columns, the annual estimation data from the WGI indicators were selected by transposing from rows to communes 1068 subsets of 17 data to complete 18,156 WGI indicator data. Storing a total of 3026 records (54,468 data) in an SPSS version 29 file adding the label metadata.

Table 2 shows the statistics describing the 18 indicators with 3026 records reported in this Global dataset, the variations in sample size (N) are due in the case of the FSI indicators to the countries not reported in the first edition (2006) and the differences for the WGI indicators to the South Sudan data starting from 2011 given the recent constitution at that date of this new country.

**Table 2:** Descriptive statistics.

Indicators	N	Minimum	Maximum	Mean	Skewness		Kurtosis	
	Statistic	Statistic	Statistic	Statistic	Statistic	Error estándar	Statistic	Error estándar
1. C1	2989	0.3	10.0	5.623	−0.282	0.045	−0.733	0.090
2. C2	2989	0.7	10.0	6.316	−0.580	0.045	−0.713	0.090
3. C3	2989	0.3	10.0	5.971	−0.111	0.045	−0.646	0.090
4. E1	2989	1.0	10.0	5.708	−0.191	0.045	−0.640	0.090
5. E2	2989	0.5	10.0	6.150	−0.557	0.045	−0.545	0.090
6. E3	2989	0.4	10.0	5.540	−0.439	0.045	−0.707	0.090
7. P1	2989	0.2	10.0	6.173	−0.679	0.045	−0.510	0.090
8. P2	2989	0.6	10.0	5.617	−0.180	0.045	−1.080	0.090
9. P3	2989	0.3	10.0	5.774	−0.349	0.045	−0.874	0.090
10. S1	2989	0.7	10.0	6.039	−0.269	0.045	−0.951	0.090
11. S2	2989	0.4	10.0	5.034	0.269	0.045	−0.951	0.090
12. X1	2989	0.3	10.0	5.699	−0.343	0.045	−0.628	0.090
13. G1	3021	−2.3	2.8	−0.136	−0.065	0.045	−0.969	0.089
14. G2	3021	−3.3	1.6	−0.165	−0.582	0.045	−0.107	0.089
15. G3	3021	−2.4	2.5	−0.106	0.345	0.045	−0.544	0.089
16. G4	3021	−2.6	2.3	−0.098	0.163	0.045	−0.535	0.089
17. G5	3021	−2.6	2.1	−0.148	0.436	0.045	−0.523	0.089
18. G6	3021	−1.9	2.5	−0.120	0.717	0.045	−0.313	0.089
N valid (per list)	2989							

**Fig. 1:**
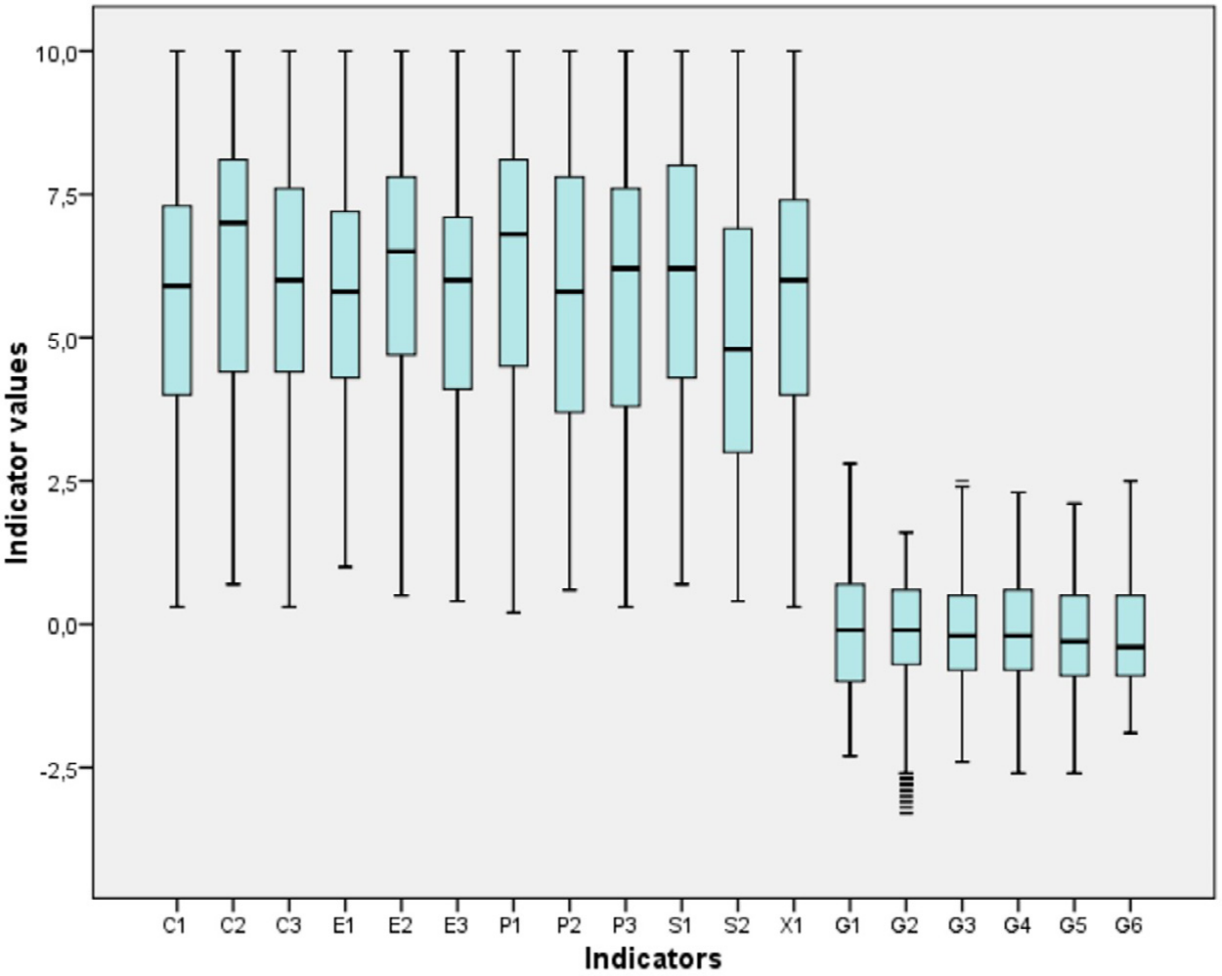
Indicators boxplot.

Fig. 1 shows in a box-and-whisker plot (boxplot) the quantitative distribution of the data for the 18 indicators reported, facilitating the comparison between the variables, highlighting their minimum and maximum values at the ends of the whiskers, the median indicated inside the box and the outliers presented by the G2 (lower) and G3 (upper) indicators. It is necessary to point out that the low values in the FSI indicators are better than the high values, and on the contrary for the WGI indicators the low values (negative) are worse than the high values (positive).

Table 3 shows the bivariate correlations, all significant, between the indicators/variables from both sources (FSI [1] and the WGI [2]), indicating the importance of jointly studying state fragility and state governance.

**Table 3:** Bivariate correlations WGI and FSI indicators.

Stats		C1	C2	C3	E1	E2	E3	P1	P2	P3	S1	S2	X1
**G1**	Pearson correl.	−.717⁎⁎	−776⁎⁎	−.602⁎⁎	−.551⁎⁎	−.584⁎⁎	−.462⁎⁎	−.865⁎⁎	−.625⁎⁎	−.903⁎⁎	−.625⁎⁎	−.584⁎⁎	−.575⁎⁎
	Signif. (bilater.)	0.000	0.000	.000	.000	.000	.000	0.000	0.000	0.000	0.000	.000	.000
	N	2989	2989	2989	2989	2989	2989	2989	2989	2989	2989	2989	2989
**G2**	Pearson correl.	−.830⁎⁎	−.751⁎⁎	−.776⁎⁎	−.591⁎⁎	−.603⁎⁎	−.525⁎⁎	−.714⁎⁎	−.680⁎⁎	−.735⁎⁎	−.651⁎⁎	−.753⁎⁎	−.636⁎⁎
	Signif. (bilater.)	0.000	0.000	0.000	.000	.000	.000	0.000	0.000	0.000	0.000	0.000	0.000
	N	2989	2989	2989	2989	2989	2989	2989	2989	2989	2989	2989	2989
**G3**	Pearson correl.	−.815⁎⁎	−.779⁎⁎	−.583⁎⁎	−.824⁎⁎	−.746⁎⁎	−.729⁎⁎	−.827**	−.871⁎⁎	−.773⁎⁎	−.803⁎⁎	−.665**	−.770⁎⁎
	Signif. (bilater.)	0.000	0.000	.000	0.000	0.000	0.000	0.000	0.000	0.000	0.000	0.000	0.000
	N	2989	2989	2989	2989	2989	2989	2989	2989	2989	2989	2989	2989
**G4**	Pearson correl.	−.793⁎⁎	−.769**	−.566⁎⁎	−.787⁎⁎	−.712⁎⁎	−.697⁎⁎	−.824**	−.818⁎⁎	−.800**	−.767⁎⁎	−.622⁎⁎	−.741⁎⁎
	Signif. (bilater.)	0.000	0.000	.000	0.000	0.000	0.000	0.000	0.000	0.000	0.000	0.000	0.000
	N	2989	2989	2989	2989	2989	2989	2989	2989	2989	2989	2989	2989
**G5**	Pearson correl.	−.848**	−.800⁎⁎	−.632⁎⁎	−.774⁎⁎	−.744⁎⁎	−.719⁎⁎	−.859**	−.842⁎⁎	−.820⁎⁎	−.777⁎⁎	−.662⁎⁎	−.741⁎⁎
	Signif. (bilater.)	0.000	0.000	0.000	0.000	0.000	0.000	0.000	0.000	0.000	0.000	0.000	0.000
	N	2989	2989	2989	2989	2989	2989	2989	2989	2989	2989	2989	2989
**G6**	Pearson correl.	−.813⁎⁎	−.789⁎⁎	−.638⁎⁎	−.725⁎⁎	−.710⁎⁎	−.685⁎⁎	−.853⁎⁎	−.803⁎⁎	−.803⁎⁎	−.736⁎⁎	−.642⁎⁎	−.696⁎⁎
	Signif. (bilater.)	0.000	0.000	0.000	0.000	0.000	0.000	0.000	0.000	0.000	0.000	0.000	0.000
	N	2989	2989	2989	2989	2989	2989	2989	2989	2989	2989	2989	2989

⁎⁎ The correlation is significant at the 0.01 level (bilateral).

## Limitations

Limitations to the reported data set are given by the lowest year reported for the FSI indicators (2006) and the latest year reported to date for the WGI indicators (2022), this limitation can be improved over time by updating the dataset using the extraction and curation protocol that we have detailed in 6 steps. Additionally, for the WGI indicators, only the estimated data has been considered, excluding the standard error, number of data sources, percentile rank, lower bound (90% confidence interval), and upper bound (90% confidence interval), to make both databases homogeneous.

## Ethics Statement

The authors were carried out in conformity with the Declaration of Helsinki. The authors have read and follow the ethical requirements for publication in Data in Brief and confirming that the current work does not involve human subjects, animal experiments, or any data collected from social media platforms. The use Fragile States Index data do not require special permission for non-commercial use [1], and the World Bank dataset is classified as Public under the Access to Information Classification Policy [2].

## Funding

A.V.-M., and G.S.-S. All authors have read and agreed to the published version of the manuscript.

## CRediT authorship contribution statement

**Alejandro Vega-Muñoz:** Conceptualization, Methodology, Data curation, Writing – original draft, Writing – review & editing, Project administration. **Paloma González-Gómez-del-Miño:** Conceptualization, Writing – review & editing, Supervision. **Guido Salazar-Sepúlveda:** Formal analysis, Data curation, Writing – original draft.

## Data Availability

Data Global Governance and Fragility (Original data) (zenodo)
